# Understanding NHS hospital admissions in England, Scotland and Wales: data linkage to the King's College Military Cohort Study

**DOI:** 10.23889/ijpds.v1i1.293

**Published:** 2017-04-19

**Authors:** Daniel Leightley, Zoe Chui, Laura Goodwin

**Affiliations:** King's Centre for Military Health Research

## Objectives

Secondary health systems in the United Kingdom (UK) are unique for recording Outpatient, Inpatient and Accident & Emergency (A&E) visits in the form of electronic health (eHealth) records. Linking regional healthcare datasets is a problematic, further challenging when linking externally, such as to the King's College Military Cohort Study (KCMCS). We introduce our methodology used for eRecord linkage. 

## Approach

eHealth records from England, Scotland and Wales offer a variety of parameters such as admission/discharge date, diagnosis, treatment/procedure undertaken and the cost of treatment. To acquire eHealth records, unique patient identifiers: NHS number, forename, surname, sex and date of birth extracted from KCMCS were provided to each region. The KCMCS contains self-reported questionnaire results for 9,990 serving/ex-serving military personal, 8,602 participants consented to linkage. eHealth records prepared for linkage in two stages. First, admission and discharge date were checked to ensure a valid date. Second, episodes were checked for consistency, ensuring that no records for individual participants were duplicated. Data available varied based on the region, this disparity between regions can result in data type variation. Hence, linkage was performed on mutual variables to ensure a uniform admission history. Creation of the linked dataset was as follows. First, records and episodes relating to an individual were brought together, to create a personal admission history. Secondly, personal admission history were linked to the KCMCS.

## Results

Linking to regional health datasets is not without its challenges. England, Scotland and Wales obtain, store and process eHealth records using different methodologies. A total of 6,336 (76.66%) participants were matched by regional health providers, with a total of 61,558 eHealth records. A total of 187 eHealth records were identified and discounted from linkage due to failure to meet criteria listed above. Verifying diagnoses completeness, Inpatient admissions were consistently code, with full completeness. Conversely, Outpatient admissions were poorly coded with 98% lacking any type of diagnosis. In addition, A&E records were sparsely coded; we identified four different regional and local coding systems to identify reason for admission. The eHealth records show promise for identifying health traits of the military. However, further work is required to identify synergy and overcome regional variations.

## Conclusion

Linkage techniques provide new opportunities for exploring the health of serving and veteran population. However, quality of identifier and linkage error are still of major concern. Further, record completeness, diagnoses accuracy and data cleaning impact the data quality.

